# Supplementary material for: Single-label and multi-label classification for disease recognition with special consideration of comorbidities
Source: BMC Med Res Methodol. 2026 Jul 10;26:155. doi: 10.1186/s12874-026-02934-w (PMC13355337; doi:10.1186/s12874-026-02934-w)
Supplement: Supplementary file 1 — Additional file 1. Additional information on the dataset used for analyses and on the results of the case study is provided in the Supplementary Material. [file 12874_2026_2934_MOESM1_ESM.pdf]

Supplementary Material for 'Single-label and Multi-label Classification for Disease Recognition with Special Consideration of Comorbidities'

Sophie Schmiegel, Hannah Marchi, Marvin-Hendrik Röchter, Martin Rudwaleit, Christiane Fuchs

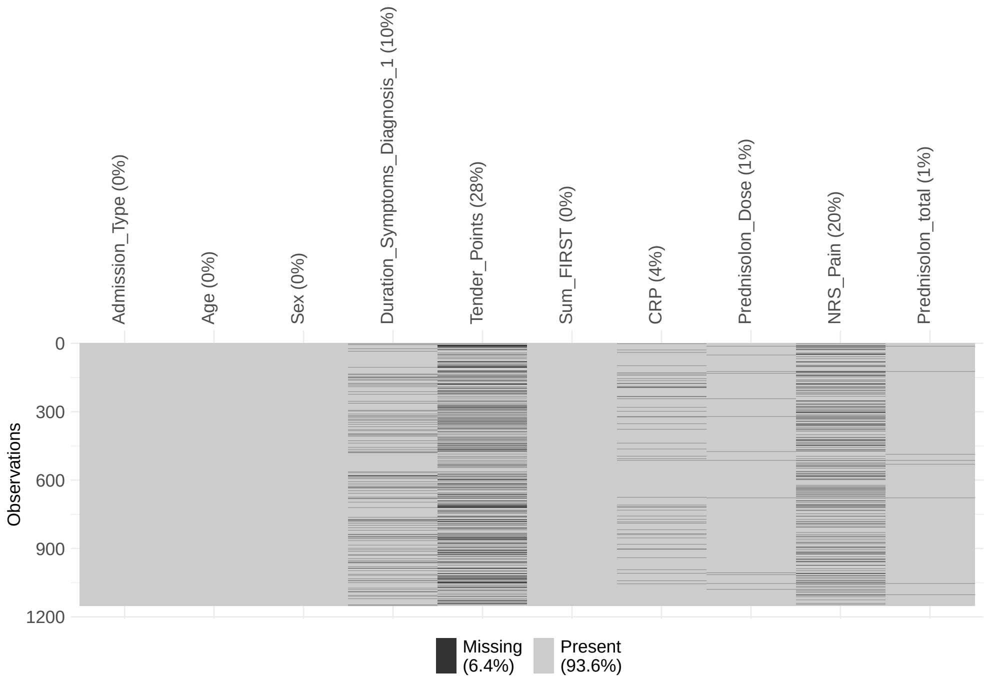

Fig. A1 Frequencies of missing values in those variables that were used for statistical analysis. The set of 1150 patients was reduced to the complete cases, resulting in 663 patients.

Table A1 True labelsets and their numbers of occurrence in the dataset under consideration. y_i^1 corresponds to FMS, y_i^2 to OPCD, y_i^3 to OA and y_i^4 to IMID.

Labelsets (*FMS, OPCD, OA, IMID*)	Number of occurrences
$(0, 1, 0, 0)$	160
$(1, 0, 0, 0)$	103
$(0, 0, 0, 1)$	100
$(0, 0, 1, 0)$	43
$(0, 1, 0, 1)$	40
$(0, 0, 1, 1)$	36
$(1, 0, 1, 0)$	32
$(1, 1, 0, 0)$	31
$(1, 0, 0, 1)$	30
$(0, 1, 1, 0)$	22
$(1, 0, 1, 1)$	21
$(1, 1, 0, 1)$	17
$(1, 1, 1, 0)$	15
$(0, 1, 1, 1)$	13
$(1, 1, 1, 1)$	0
$(0, 0, 0, 0)$	0

Table A2 p-values and adjusted p-values obtained by Pearson's χ^2-test.

	p-value	adj. p-value
IMID and FMS	$3.98e^{-6}$	0.00
IMID and OA	$9.93e^{-1}$	1.00
IMID and OPCD	$5.46e^{-13}$	0.00
FMS and OA	1.00	1.00
FMS and OPCD	$5.90e^{-15}$	0.00
OA and OPCD	$4.34e^{-8}$	0.00

Table A3 IMID-specific performance (averaged across ten CV folds; 95 % confidence intervals are given in brackets) of the six described approaches for recognizing IMID. The approach as well as the considered classes are provided in the column names. The highest average measures per approach are marked with *, the highest average measure per classifier (DT, RF, LRM, k-NN, MLP) is written in bold. A graphical representation of the results is provided in Figure 4 in the main manuscript.

	SLC with $C = 2$ {*IMID*, *noIMID*}		SLC with $C = 4$ {*IMID*, *FMS**, *OA**, *OPCD**}		MLC with $C = 4$ {*IMID*, *FMS*, *OA*, *OPCD*}	
	Approach 1a	Approach 1b	Approach 2a	Approach 2b	Approach 3a	Approach 3b
DT						
Sensitivity	0.511 [0.288; 0.734]	**0.568** [0.481; 0.654]	0.522 [0.282; 0.761]	0.357 [0.153; 0.560]	0.477 [0.333; 0.620]	0.528 [0.341; 0.715]
Specificity	0.849 [0.797; 0.901]	0.776 [0.639; 0.912]	0.719 [0.505; 0.933]	**0.854** [0.784; 0.924]	0.852 [0.783; 0.920]	0.686 [0.499; 0.872]
Precision	**0.673** [0.530; 0.815]	0.627 [0.511; 0.743]	0.553 [0.381; 0.725]	0.588 [0.323; 0.852]	0.670 [0.559; 0.780]	0.522 [0.358; 0.686]
F1-score	0.575 [0.392; 0.758]	**0.591** [0.548; 0.634]	0.521 [0.351; 0.690]	0.437 [0.221; 0.654]	0.552 [0.441; 0.664]	0.515 [0.398; 0.632]
RF						
Sensitivity	0.501 [0.367; 0.636]	**0.629*** [0.488; 0.770]	0.625* [0.492; 0.758]	0.459* [0.282; 0.637]	0.516 [0.345; 0.687]	0.565 [0.425; 0.706]
Specificity	0.870 [0.789; 0.951]	0.795* [0.717; 0.873]	0.785* [0.687; 0.883]	**0.897** [0.849; 0.945]	0.867* [0.812; 0.922]	0.800* [0.722; 0.877]
Precision	0.708 [0.537; 0.879]	0.658* [0.524; 0.793]	0.648* [0.488; 0.808]	**0.735** [0.610; 0.860]	0.706* [0.569; 0.842]	0.640* [0.521; 0.760]
F1-score	0.580 [0.472; 0.687]	**0.639*** [0.535; 0.742]	0.632* [0.518; 0.746]	0.558* [0.419; 0.696]	0.591* [0.447; 0.735]	0.594 [0.507; 0.681]
LRM						
Sensitivity	0.521 [0.378; 0.663]	0.616 [0.442; 0.789]	0.597 [0.443; 0.750]	0.438 [0.223; 0.653]	0.523 [0.327; 0.718]	**0.623*** [0.468; 0.778]
Specificity	0.883* [0.831; 0.935]	0.790 [0.713; 0.866]	0.765 [0.691; 0.839]	**0.904*** [0.842; 0.965]	0.861 [0.781; 0.942]	0.743 [0.659; 0.826]
Precision	0.735* [0.631; 0.839]	0.648 [0.551; 0.745]	0.616 [0.557; 0.675]	**0.740*** [0.611; 0.868]	0.702 [0.552; 0.852]	0.604 [0.511; 0.698]
F1-score	0.604 [0.507; 0.701]	**0.624** [0.541; 0.707]	0.601 [0.525; 0.678]	0.540 [0.359; 0.721]	0.590 [0.450; 0.729]	0.607* [0.539; 0.675]
k-NN						
Sensitivity	0.450 [0.319; 0.580]	0.556 [0.456; 0.655]	0.511 [0.340; 0.682]	0.372 [0.237; 0.506]	0.445 [0.259; 0.630]	**0.581** [0.462; 0.701]
Specificity	0.822 [0.684; 0.960]	0.663 [0.513; 0.813]	0.769 [0.651; 0.887]	**0.865** [0.733; 0.997]	0.808 [0.675; 0.940]	0.600 [0.500; 0.699]
Precision	0.629 [0.418; 0.840]	0.517 [0.400; 0.633]	0.584 [0.433; 0.736]	**0.652** [0.498; 0.805]	0.600 [0.413; 0.786]	0.479 [0.382; 0.576]
F1-score	0.511 [0.423; 0.599]	0.532 [0.453; 0.610]	**0.542** [0.388; 0.696]	0.468 [0.339; 0.598]	0.501 [0.358; 0.644]	0.522 [0.444; 0.600]
MLP						
Sensitivity	0.533* [0.398; 0.668]	0.556 [0.421; 0.692]	0.582 [0.464; 0.701]	0.458 [0.298; 0.617]	0.550* [0.386; 0.714]	**0.597** [0.426; 0.768]
Specificity	**0.877** [0.822; 0.933]	0.690 [0.564; 0.815]	0.735 [0.573; 0.898]	0.785 [0.670; 0.899]	0.781 [0.702; 0.861]	0.664 [0.526; 0.801]
Precision	**0.732** [0.635; 0.829]	0.535 [0.409; 0.661]	0.590 [0.438; 0.742]	0.576 [0.436; 0.716]	0.613 [0.504; 0.721]	0.530 [0.377; 0.683]
F1-score	**0.612*** [0.517; 0.706]	0.539 [0.456; 0.623]	0.580 [0.496; 0.664]	0.504 [0.386; 0.622]	0.576 [0.451; 0.701]	0.555 [0.429; 0.681]

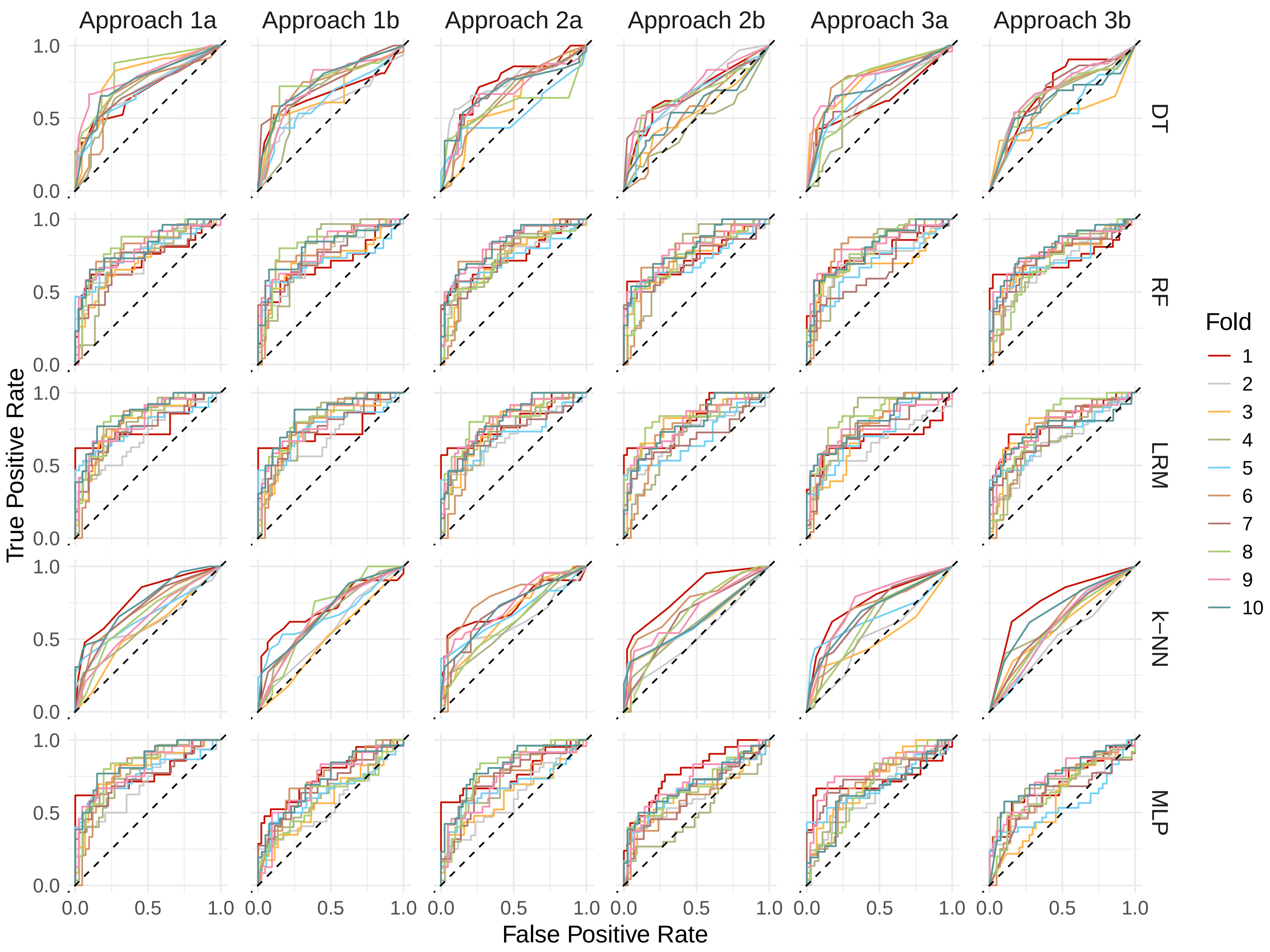

Fig. A2 Receiver operating characteristic curves for each of the six approaches and each classifier. The different folds of nested CV are displayed in different colors.

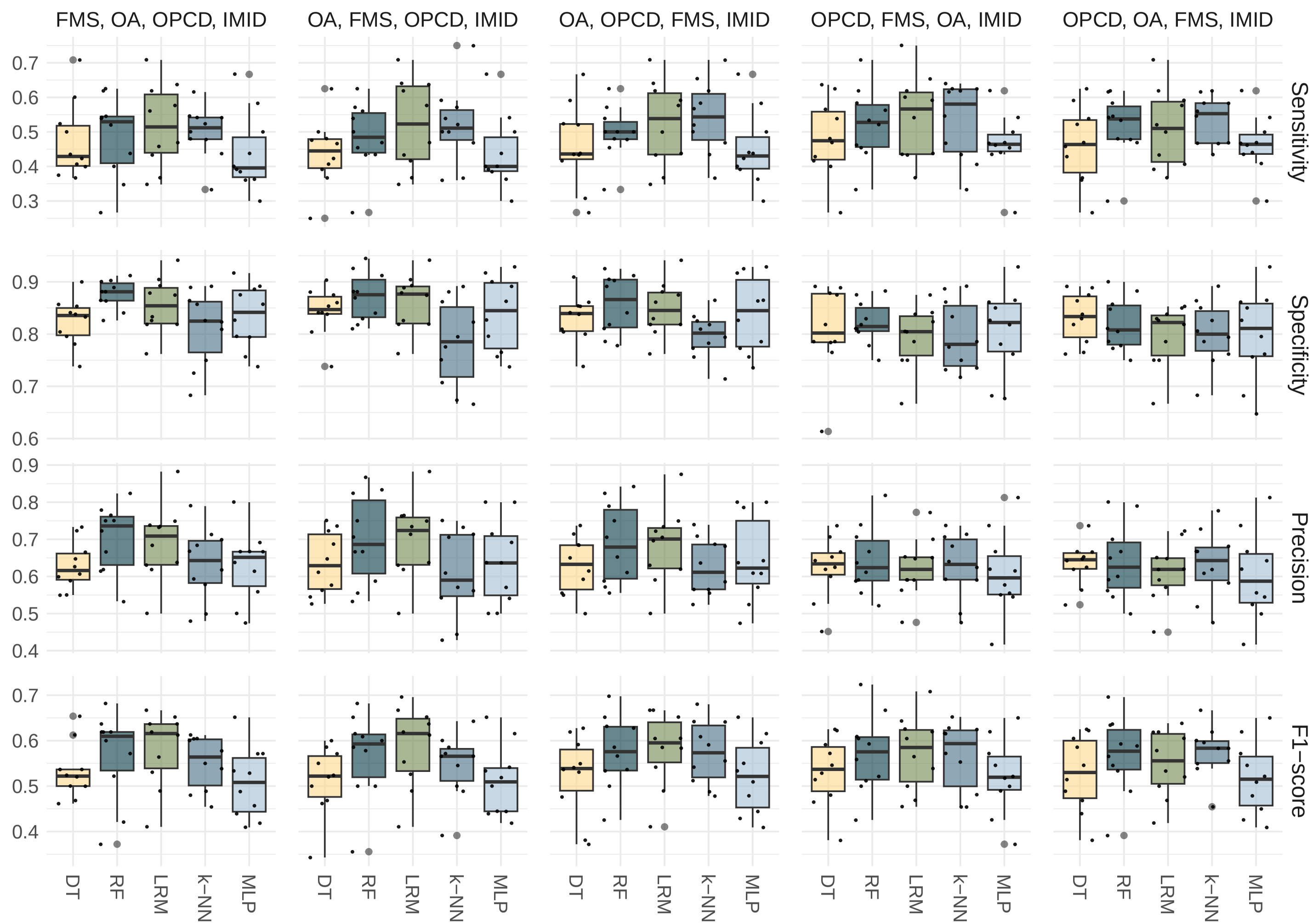

Fig. A3 Performance of MLC models for five out of the six label orderings ending with IMID. The sixth one (FMS, OPCD, OA, IMID) leads to the highest average F1-score and is shown in Figure 4 in the main manuscript.

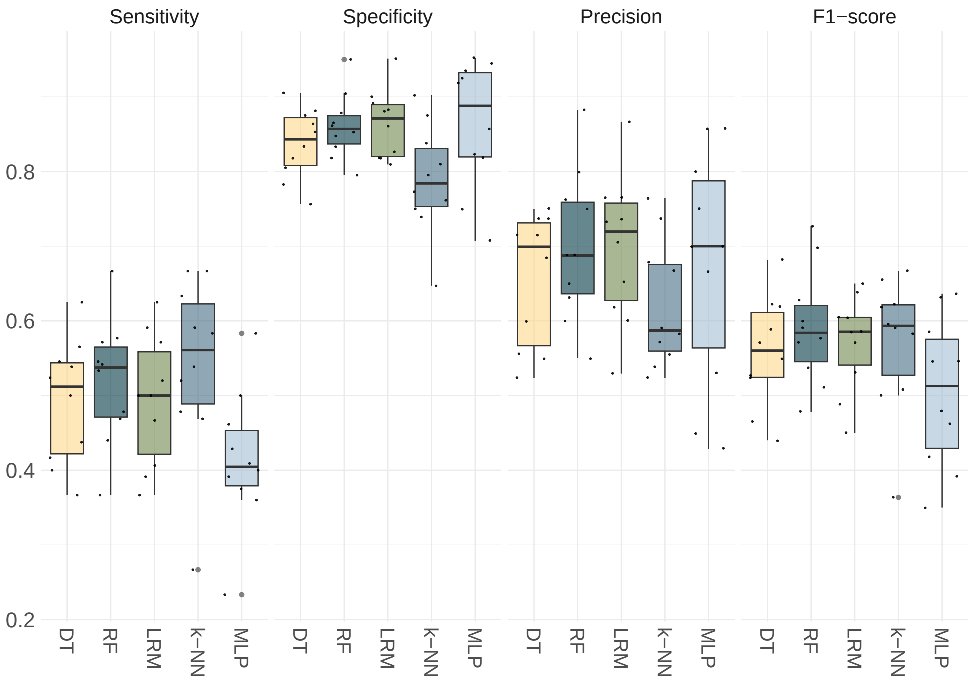

Fig. A4 Performance of the MLC approaches with $C = 3$ classes, namely *FMS*, *OPCD* and *IMID*.
